# Removal of Vessical Calculi, the Nucleus of Which Was a Pistol Bullet

**Published:** 1869-02

**Authors:** J. C. Hughes

**Affiliations:** Professor of Surgery, Medical Department Iowa State University


### REMOVAL OF VESICAL CALCULI,

*(The nucleus of which was a Pistol Bullet.)*

BY J. C. HUGHES, M.D.

Professor of Surgery, Medical Department Iowa State University.

JAMES MITCHELL, aged thirty-four, a native of Aberdeenshire, Scotland, while engaged in the freighting business between Leavenworth and Denver City, when near Cottonwood, in the month of April, 1864, was accidentally shot by one of the conductors of the train. The ball entered the posterior portion of the pelvis, near the left sacro-illiac juncture, and lodged within the viscera of its cavity. Mr. M. was placed under the professional care of my friend, Dr. LaForce, surgeon of the Seventh Iowa cavalry, and at the time surgeon-in-charge at Ft. Cottonwood. I have not the statement from the doctor, but the patient informs me that during his six weeks confinement in hospital he suffered great pain in the lower portion of rectum, which was supposed to have been injured, and that difficulty and suffering also attended his efforts to urinate. He so far recovered as to be able to render the surgeon some assistance about the fort; yet his suffering at times was very severe, giving evidence of increased urinary trouble. In October the doctor sent the patient to his home in Iowa; and in June, 1865, finding that his symptoms gave increased evidence of the presence of urinary calculi, he brought him to Keokuk for examination and treatment. Within a fortnight I examined the patient four different times, each time most carefully sounding the bladder and rectum, but was unable to find the foreign body. Feeling assured that the bullet was the cause of the trouble, and that if not loose within the viscus must be lodged within its walls, the question of an exploring operation, in order to relieve the patient's suffering, was discussed, but a negative decision arrived at. The patient returned home, and was lost sight of until February, 1868, when I received a letter from the doctor, stating that Mr. Mitchell's sufferings had become so severe that life was a burden to him, and that his only hope was from operation. He again visited me, and upon the introduction of the sound I at once discovered a large stone. The bladder and rectum having suffered most seriously from inflammation; thickening ulceration and adhesions existed, and the pain which he suffered so excruciating as to require him from thirty to sixty minutes to make the change from the erect to the horizontal position.

Saturday morning, February 22, the patient was brought before the medical class of the Iowa University for operation. When fully under the influence of chloroform I proceeded to perform the bilateral operation with the double lithotom cache of DuPuytren. The incision was as large as safety to the pudic arteries would justify. Yet I found the stone too large for the opening, and proceeded with the lithotripter to crush, which I succeeded in accomplishing without great difficulty. On the removal of the fragments I found the bullet, which had formed the nucleus of the stone. The calculi was of the phosphatic variety, and of the size of a large hen-egg. This patient, although recovering, has not made so favorable a cure as many others which I have operated upon. I attribute this not to any fault in the treatment, but to the fact that there exists a tubercular diathesis. The disease, in connection with the rectum and bladder, left sinuses, which have resulted in recto-vesical fistula, the principal opening connecting within the sphincter ani. This condition militates largely against complete recovery. To close the fistula we may induce increased pulmonary trouble. Yet the nature of the case, and his present condition would seem to justify the effort, and I therefore have placed him under treatment with the hope of relief.

